# Impact of COVID-19 therapeutics on the development of post-infectious lung fibrosis

**DOI:** 10.3389/fmicb.2025.1677734

**Published:** 2025-11-06

**Authors:** Yong Jun Choi, Ji Eun Nam, Chul Hwan Park, Ji Ye Jung, Eun Hye Lee, Hye Jung Park, Chi Young Kim, Jae Hwa Cho, Min Kwang Byun

**Affiliations:** 1 Division of Pulmonary and Critical Care Medicine, Department of Internal Medicine, Gangnam Severance Hospital, Yonsei University College of Medicine, Seoul, Republic of Korea; 2 Department of Radiology and the Research Institute of Radiological Science, Gangnam Severance Hospital, Yonsei University College of Medicine, Seoul, Republic of Korea; 3 Division of Pulmonary and Critical Care Medicine, Department of Internal Medicine, Severance Hospital, Yonsei University College of Medicine, Seoul, Republic of Korea; 4 Division of Pulmonology, Allergy and Critical Care Medicine, Department of Internal Medicine, Yongin Severance Hospital, Yonsei University College of Medicine, Yongin, Republic of Korea

**Keywords:** post-COVID-19 pulmonary fibrosis, remdesivir, baricitinib, COVID-19 pandemic, SARS-CoV-2 infection, computed tomography

## Abstract

**Background:**

Post-COVID-19 pulmonary fibrosis (PCPF) is a significant long-term complication in survivors of COVID-19. In this study, we aimed to identify clinical risk factors for PCPF and evaluate the impact of COVID-19–related therapies.

**Methods:**

We retrospectively studied hospitalized adults with confirmed COVID-19 across three hospitals in South Korea from 2020 to 2022. Inclusion required chest computed tomography (CT) imaging both before and after COVID-19 infection. PCPF was defined as fibrotic changes seen on follow-up CT performed at least one month after recovery.

**Results:**

Among 5,720 hospitalized adults with COVID-19, 688 met the inclusion criteria, and 87 (12.6%) developed PCPF based on follow-up CT. In the multivariate logistic regression, pre-existing renal disease (adjusted odds ratio [aOR] 3.287; 95% confidential interval [CI]: 1.260–8.580; *p* = 0.014), higher hemoglobin levels (aOR: 1.194; 95% CI: 1.032–1.387; *p* = 0.018) and elevated CRP (aOR: 1.005; 95% CI: 1.001–1.009; *p* = 0.022) were independently associated with increased risk of PCPF. Remdesivir use was significantly associated with a reduced risk of PCPF (aOR: 0.359; 95% CI: 0.176–0.734; *p* = 0.005), whereas baricitinib use was associated with an increased risk (aOR: 5.633; 95% CI: 1.642–19.548; *p* = 0.006).

**Conclusion:**

PCPF remains a relevant sequela in COVID-19 survivors. Remdesivir and baricitinib use were associated with a reduced and increased risk of PCPF, respectively. Although adjusted for multiple confounders, residual indication bias of each treatment cannot be completely excluded. Therefore, prospective studies are needed to validate these associations.

## Introduction

The coronavirus disease 2019 (COVID-19) pandemic, caused by severe acute respiratory syndrome coronavirus 2 (SARS-CoV-2) infection, has imposed an unprecedented global burden over the past five years, including social, economic, and healthcare system disruptions worldwide (Nicola et al., 2020; Fink et al., 2022; Choi et al., 2023; Heo et al., 2025). While the acute manifestations of COVID-19 range from mild respiratory symptoms to severe pneumonia and acute respiratory distress syndrome, accumulating evidence suggests that the disease may also result in chronic symptoms and long-term complications affecting multiple organ systems (Lopez-Leon et al., 2021; Desai et al., 2022; Li et al., 2023; Lee et al., 2024). Among these, post-COVID-19 pulmonary fibrosis (PCPF) has emerged as a significant respiratory sequela, particularly in patients who experienced severe or prolonged illness.

PCPF is defined as persistent fibrotic alterations in the lung parenchyma following SARS-CoV-2 infection. It is radiologically characterized by interstitial thickening, reticulation, traction bronchiectasis, architectural distortion, and occasionally honeycombing, often accompanied by impaired lung function (George et al., 2020; Li et al., 2022; Yoon and Uh, 2022). Although the prevalence and trajectory of these fibrotic changes vary, a subset of patients continue to demonstrate radiologic and functional abnormalities for months after recovery.

Despite growing interest in therapeutic strategies to prevent or mitigate PCPF, pharmacologic strategies for preventing or treating post-COVID fibrosis remain largely undefined. Most pharmacologic interventions under investigation have focused on antifibrotic agents, such as nintedanib or pirfenidone; however, evidence supporting their routine use in post-COVID settings remains limited (George et al., 2020; Shu et al., 2024). Evaluating the role of antiviral agents in this context poses further challenges, because their indications are often closely tied to the severity of illness or underlying risk factors. Moreover, baseline laboratory findings and comorbidities of patients can influence both treatment decisions and long-term outcomes, confounding the analysis of direct drug effects.

In this study, we aimed to identify key risk factors contributing to PCPF by conducting a comprehensive analysis that adjusts for demographic characteristics, comorbid conditions, laboratory parameters, and illness severity. This approach seeks to clarify independent predictors of fibrotic sequelae and inform future preventive and therapeutic strategies.

## Materials and methods

### Study design and patients

**FIGURE 1:**
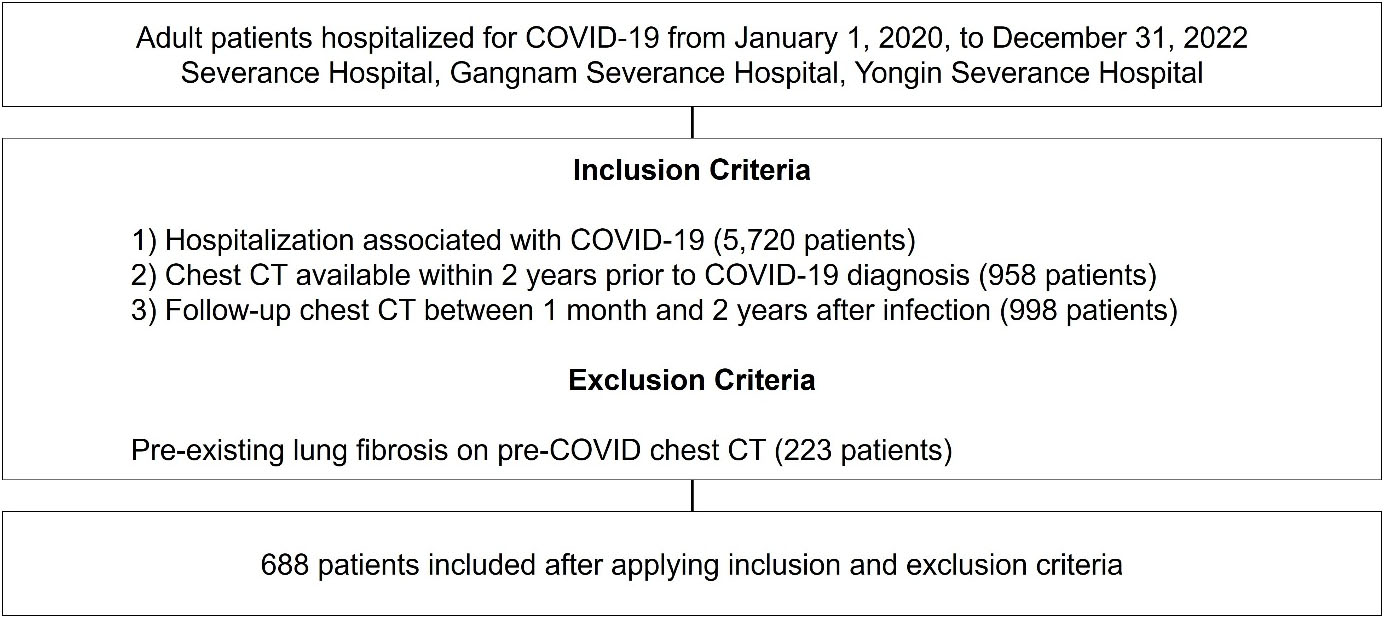
Study flow chart. COVID-19, coronavirus disease 2019; CT, computed tomography.

This retrospective study was conducted at three tertiary hospitals in South Korea–Severance Hospital (2,499 beds), Gangnam Severance Hospital (824 beds), and Yongin Severance Hospital (708 beds). Patients were screened from 1 January 2020, to 31 December 2022 and included in this study if they met all of the following criteria (Figure 1): (1) A confirmed diagnosis of COVID-19 based on a positive reverse transcription polymerase chain reaction (RT-PCR) test for SARS-CoV-2 and hospitalized due to COVID-19–related illness, indicating moderate to critical disease requiring inpatient care; (2) availability of a chest computed tomography (CT) scan performed within two years prior to the COVID-19 diagnosis; and (3) a follow-up chest CT scan conducted between one month and two years after COVID-19 infection, enabling the assessment of post-infectious pulmonary changes. Patients with evidence of pre-existing lung fibrosis on chest CT scans performed prior to their COVID-19 diagnosis were excluded from the study.

### Data collection

All information was extracted from the electronic health records, including patient demographics, clinical data, such as vital signs, blood tests, admission and discharge diagnoses, code status, and medication. For patients who experienced more than one episode of COVID-19 infection, data collection was based on the first documented episode. Comorbidities were categorized and quantified using the Charlson Comorbidity Index (CCI), based on the updated algorithm by Quan et al. (2005), which incorporates International Classification of Diseases (ICD) codes to systematically score 17 comorbid conditions.

All chest CT images were independently reviewed by two board-certified thoracic radiologists—JEN, with over 30 years of experience, and CHP, with 20 years of experience. In cases of disagreement, fibrosis was determined through interobserver consensus to ensure consistency. Interobserver agreement was high, with a Cohen’s kappa coefficient (κ) of 0.81.

### Definitions of clinical outcomes and variables

The primary clinical outcome was the development of pulmonary fibrosis. Pulmonary fibrosis was defined based on radiologic features, including reticulation, traction bronchiectasis, architectural distortion, and/or honeycombing, as observed on high-resolution CT scans (Piciucchi et al., 2016; George et al., 2020; Li et al., 2022; Yoon and Uh, 2022; Lederer et al., 2024). To distinguish post-infectious fibrotic changes from acute-phase findings such as ground-glass opacities (GGO), lung fibrosis was confirmed based on chest CT performed at least one month after recovery from the acute phase of COVID-19. Cases with fibrotic features observed only on CT scans taken during the acute phase were not considered definitive for PCPF.

COVID-19 severity was defined according to established criteria from the World Health Organization and the US National Institutes of Health (Guerin et al., 2022). Mild to moderate disease was characterized by the presence of respiratory symptoms or radiographic evidence of pneumonia without hypoxemia (SpO_2_ ≥ 94%) or the need for supplemental oxygen. Severe disease was defined by signs of respiratory distress, including a respiratory rate > 30 breaths per minute or SpO_2_ < 94% on room air, requiring oxygen therapy. Critical disease was defined by life-threatening complications, including acute respiratory distress syndrome (ARDS), septic shock, or multiorgan dysfunction, necessitating intensive care interventions such as mechanical ventilation or extracorporeal membrane oxygenation (ECMO). In addition, concurrent infections were defined to include co-infections at baseline as well as secondary infections that developed during hospitalization.

### Statistical analysis

We analyzed the demographic and clinical characteristics of patients in the event (lung fibrosis) and non-event groups using chi-square tests for categorical variables. For continuous variables, either independent *t*-tests or Wilcoxon rank-sum tests were applied, depending on the normality of the data distribution.

Logistic regression analysis was performed to identify potential risk factors associated with the development of post-COVID lung fibrosis. Multicollinearity was evaluated using the variance inflation factor (VIF), and variables with VIF > 5.0 were excluded from the multivariate model. All statistical analyses were conducted using R (version 4.4.0). A two-tailed *p*-value < 0.05 was considered statistically significant.

### Ethics

This study was approved by the Institutional Review Board (IRB) of the Gangnam Severance Hospital (Approval number: 3-2023-0428). The data were collected following the principles of the Declaration of Helsinki. All data for the retrospective analysis of the clinical outcomes were fully anonymized before access, and the ethics committee waived the requirement for written informed consent because of the retrospective nature of this study.

## Results

### Baseline characteristics

Over three years, 5,720 adult patients were hospitalized for COVID-19-related conditions across the three institutions. Among these, 688 patients met the inclusion criteria and were included in the final analysis. Of these, 87 (12.6%) were identified as having developed PCPF based on follow-up chest CT findings.

The baseline characteristics of the study population are presented in Table 1. Compared to the non-PCPF group, patients in the PCPF group had significantly higher body weight (median 63.1 kg [interquartile range, IQR: 55.4–71.2] vs. 61.0 kg [IQR: 52.5–68.5], *p* = 0.035) and body mass index (BMI) (median 24.0 [IQR: 21.8–25.5] vs. 22.8 [IQR: 20.2–25.4], *p* = 0.034). The prevalence of solid tumors was significantly lower in the PCPF group than in the non-PCPF group (48.2% vs. 61.3%, *p* = 0.033). Similarly, metastatic tumors were also less frequent in the PCPF group (14.5% vs. 25.1%, *p* = 0.047). Except for malignancy-related conditions, other comorbidities such as cardiovascular, pulmonary, metabolic, hepatic, and neurologic diseases showed no significant differences between the two groups (all *p* > 0.05).

**TABLE 1:** Baseline characteristics.

	Total (*N* = 688)	Non-PCPF (*N* = 601)	PCPF (*N* = 87)	*P*-value
Age (years)	67.0 [57.0; 76.0]	68.0 [57.0; 77.0]	64.0 [57.5; 73.0]	0.069
**Sex**				
- Male	401 (58.3%)	346 (57.6%)	55 (63.2%)	0.378
- Female	287 (41.7%)	255 (42.4%)	32 (36.8%)	
Height (cm)	163.0 [158.0; 170.0]	163.0 [157.9; 169.8]	163.0 [158.0; 170.0]	0.656
**Weight (kg)**	**61.3 [52.8; 69.1]**	**61.0 [52.5; 68.5]**	**63.1 [55.4; 71.2]**	0.035*
**BMI (kg/m^2^)**	**23.0 [20.4; 25.4]**	**22.8 [20.2; 25.4]**	**24.0 [21.8; 25.5]**	0.034*
Never smoker	556 (80.8%)	484 (80.5%)	72 (82.8%)	0.728
**Charlson Comorbidity Index**				
Myocardial infarction	28 (4.5%)	25 (4.6%)	3 (3.6%)	0.901
Congestive heart failure	157 (25.1%)	136 (25.1%)	21 (25.3%)	1.000
Peripheral vascular disease	64 (10.2%)	52 (9.6%)	12 (14.5%)	0.243
Cerebrovascular disease	99 (15.8%)	85 (15.7%)	14 (16.9%)	0.909
Dementia	45 (7.2%)	41 (7.6%)	4 (4.8%)	0.501
Chronic pulmonary disease	175 (28.0%)	151 (27.9%)	24 (28.9%)	0.946
Rheumatic disease	22 (3.5%)	20 (3.7%)	2 (2.4%)	0.787
Peptic ulcer disease	126 (20.2%)	105 (19.4%)	21 (25.3%)	0.268
Mild liver disease	104 (16.6%)	88 (16.2%)	16 (19.3%)	0.593
Diabetes without chronic complication	207 (33.1%)	181 (33.4%)	26 (31.3%)	0.804
Diabetes with chronic complication	81 (13.0%)	72 (13.3%)	9 (10.8%)	0.659
Paraplegia and hemiplegia	32 (5.1%)	28 (5.2%)	4 (4.8%)	1.000
Renal disease	133 (21.3%)	111 (20.5%)	22 (26.5%)	0.269
**Any malignancy†**	**372 (59.5%)**	**332 (61.3%)**	**40 (48.2%)**	0.033*
Moderate or severe liver disease	18 (2.9%)	16 (3.0%)	2 (2.4%)	1.000
**Metastatic solid tumor**	**148 (23.7%)**	**136 (25.1%)**	**12 (14.5%)**	0.047*
AIDS/HIV	0 (0%)	0 (0%)	0 (0%)	1.000

PCPF, post-COVID pulmonary fibrosis; BMI, body mass index; AIDS, acquired immunodeficiency syndrome; HIV, human immunodeficiency virus. Bold and * indicate *P* < 0.05. †Including lymphoma and leukemia, except malignant neoplasm of the skin.

### Clinical severity, respiratory support, and initial laboratory findings in relation to PCPF

Significant differences were observed in clinical severity and the use of respiratory support between patients who developed PCPF and those who did not (Table 2). A notably higher proportion of patients in the PCPF group experienced critical illness (28.7% vs. 11.0%, *p* < 0.001) and required invasive mechanical ventilation (28.7% vs. 10.8%, *p* < 0.001) or ECMO (6.9% vs. 0.3%, *p* < 0.001). Additionally, the use of high-flow nasal cannula (HFNC) and conventional oxygen therapy (COT) was more frequent in the PCPF group than in the non-PCPF group (77.0% vs. 58.1%, *p* = 0.001 and 67.8% vs. 51.4%, *p* = 0.006, respectively; Table 2).

**TABLE 2:** Comparison of acute phase clinical characteristics and laboratory findings between PCPF and non-PCPF groups.

	Total (*N* = 688)	Non-PCPF (*N* = 601)	PCPF (*N* = 87)	*P*-value
**Severity**				< 0.001*
- Mild to moderate	253 (36.8%)	236 (39.3%)	17 (19.5%)	
- Severe	344 (50.0%)	299 (49.8%)	45 (51.7%)	
- Critical	91 (13.2%)	66 (11.0%)	25 (28.7%)	
**Conventional oxygen therapy**	**368 (53.5%)**	**309 (51.4%)**	**59 (67.8%)**	0.006*
**High-flow nasal cannula**	**416 (60.5%)**	**349 (58.1%)**	**67 (77.0%)**	0.001*
**Invasive mechanical ventilation**	**90 (13.1%)**	**65 (10.8%)**	**25 (28.7%)**	< 0.001*
**Extracorporeal membrane oxygenation**	**8 (1.2%)**	**2 (0.3%)**	**6 (6.9%)**	< 0.001*
**Hemoglobin (g/dL)**	**10.8 [9.2; 12.8]**	**10.6 [9.1; 12.6]**	**12.0 [9.8; 14.1]**	0.001*
White blood cells (10^3^/μL)	6.7 [4.2; 9.8]	6.7 [4.1; 9.9]	7.2 [4.6; 9.3]	0.645
Platelet (10^3^/μL)	179.0 [121.0; 243.0]	180.0 [120.0; 244.0]	165.0 [131.5; 230.0]	0.529
C-reactive protein (mg/dL)	49.5 [17.1; 109.1]	47.8 [15.9; 104.5]	62.7 [22.7; 132.8]	0.077
Aspartate transaminase (IU/L)	33.0 [23.0; 54.0]	33.0 [22.0; 52.0]	35.0 [25.5; 58.5]	0.076
**Alanine transaminase (IU/L)**	**22.0 [14.0; 38.0]**	**21.0 [13.0; 36.0]**	**30.0 [18.5; 45.5]**	0.001*
Total protein (g/dL)	6.1 ± 0.9	6.1 ± 0.9	5.9 ± 0.8	0.158
Albumin (g/dL)	3.4 [3.0; 3.9]	3.4 [3.0; 3.9]	3.4 [3.0; 3.8]	0.559
Creatinine (mg/dL)	0.8 [0.6; 1.1]	0.8 [0.6; 1.1]	0.8 [0.6; 1.0]	0.648
**Concurrent infection**				
**-Bacterial pneumonia**	**155 (22.5%)**	**127 (21.1%)**	**28 (32.2%)**	0.030*
- CNS infection	10 (1.5%)	8 (1.3%)	2 (2.3%)	0.821
- Neck infection	33 (4.8%)	27 (4.5%)	6 (6.9%)	0.476
- Abdomen infection	198 (28.8%)	172 (28.6%)	26 (29.9%)	0.907
- Biliary infection	63 (9.2%)	58 (9.7%)	5 (5.7%)	0.327
- Liver infection	22 (3.2%)	21 (3.5%)	1 (1.1%)	0.403
- Genitourinary infection	216 (31.4%)	193 (32.1%)	23 (26.4%)	0.346
- Soft tissue infection	46 (6.7%)	42 (7.0%)	4 (4.6%)	0.545

PCPF, post-COVID pulmonary fibrosis; CNS, central nervous system. Bold and * indicate *P* < 0.05.

Among laboratory markers measured during the acute phase of COVID-19, patients with PCPF had significantly higher hemoglobin levels than those without the condition (median 12.0 g/dL [IQR: 9.8–14.1] vs. 10.6 g/dL [IQR: 9.1–12.6], *p* = 0.001) and alanine aminotransferase (ALT) (median 30.0 IU/L [IQR: 18.5–45.5] vs. 21.0 IU/L [IQR: 13.0–36.0], *p* = 0.001). While C-reactive protein (CRP) and aspartate aminotransferase (AST) levels tended to be higher in the PCPF group, these differences were not statistically significant (*p* = 0.077 and *p* = 0.076, respectively).

Concurrent infections were common among the study population, most frequently involving the genitourinary tract (31.4%) and abdomen (28.8%), followed by bacterial pneumonia (22.5%). Compared with the non-PCPF group, patients with PCPF showed a significantly higher rate of bacterial pneumonia (32.2% vs. 21.1%, *p* = 0.030; Table 2). The frequencies of other infection sites, including CNS, neck, biliary, liver, and soft tissue infections, did not differ significantly between groups.

### Association between COVID-19 treatment and the development of pulmonary fibrosis

We examined the relationship between the use of COVID-19–related medications and the development of PCPF (Table 3). The overall use of systemic steroids was significantly higher in the PCPF group than in the non-PCPF group (88.5% vs. 79.2%, *p* = 0.041). Among the different types of steroids, prednisolone (65.5% vs. 41.9%, *p* < 0.001) and methylprednisolone (43.7% vs. 20.0%, *p* < 0.001) were more frequently administered to patients with PCPF, whereas no significant differences were observed in the use of dexamethasone or hydrocortisone between the two groups. Regarding antiviral therapy, remdesivir and nirmatrelvir/ritonavir (Paxlovid) showed no significant association with the development of fibrosis (*p* = 0.954 and *p* = 1.000, respectively). Notably, the use of baricitinib, a JAK inhibitor and tocilizumab, an IL-6 receptor antagonist, was higher in the PCPF group than in the non-PCPF group (9.2% vs. 2.0%, *p* = 0.001 and 17.2% vs. 4.5%, *p* < 0.001, respectively).

**TABLE 3:** Comparison of COVID-19 treatments between patients with and without PCPF.

	Total (*N* = 688)	Non-PCPF (*N* = 601)	PCPF (*N* = 87)	*P*-value
**Steroid**	**553 (80.4%)**	**476 (79.2%)**	**77 (88.5%)**	0.041*
- Dexamethasone	428 (62.2%)	369 (61.4%)	59 (67.8%)	0.300
**- Prednisolone**	**309 (44.9%)**	**252 (41.9%)**	**57 (65.5%)**	< 0.001*
**- Methylprednisolone**	**158 (23.0%)**	**120 (20.0%)**	**38 (43.7%)**	< 0.001*
- Hydrocortisone	94 (13.7%)	81 (13.5%)	13 (14.9%)	0.838
Antiviral agents	473 (68.8%)	413 (68.7%)	60 (69.0%)	1.000
- Remdesivir	437 (63.5%)	381 (63.4%)	56 (64.4%)	0.954
- Nirmatrelvir/ritonavir	27 (3.9%)	24 (4.0%)	3 (3.4%)	1.000
- Molnupiravir	12 (1.7%)	12 (2.0%)	0 (0.0%)	0.373
**Immune modulator**	**62 (9.0%)**	**39 (6.5%)**	**23 (26.4%)**	**< 0.001**
**- Baricitinib**	**20 (2.9%)**	**12 (2.0%)**	**8 (9.2%)**	0.001*
**- Tocilizumab**	**42 (6.1%)**	**27 (4.5%)**	**15 (17.2%)**	< 0.001*
**Detailed distribution of medication use**				
Steroid				0.008*
- No steroids	135 (19.6%)	125 (20.8%)	10 (11.5%)	
- Dexamethasone only	207 (30.1%)	188 (31.3%)	19 (21.8%)	
- Steroid switching (including dexamethasone)	221 (32.1%)	181 (30.1%)	40 (46.0%)	
- Other steroids	125 (18.2%)	107 (17.8%)	18 (20.7%)	
Antiviral agents				0.255
- No antivirals	215 (31.2%)	188 (31.3%)	27 (31.0%)	
- Remdesivir only	434 (63.1%)	378 (62.9%)	56 (64.4%)	
- Nirmatrelvir+ritonavir only	24 (3.5%)	21 (3.5%)	3 (3.4%)	
- Lopinavir only	1 (0.1%)	0 (0.0%)	1 (1.1%)	
- Molnupiravir only	10 (1.5%)	10 (1.7%)	0 (0.0%)	
- Remdesivir+nirmatrelvir+ritonavir	2 (0.3%)	2 (0.3%)	0 (0.0%)	
- Nirmatrelvir+ritonavir+molnupiravir	1 (0.1%)	1 (0.2%)	0 (0.0%)	
- Remdesivir+nirmatrelvir+ritonavir+molnupiravir	1 (0.1%)	1 (0.2%)	0 (0.0%)	
Immune modulators				< 0.001*
- No immunomodulators	626 (91.0%)	562 (93.5%)	64 (73.6%)	
- Baricitinib only	20 (2.9%)	12 (2.0%)	8 (9.2%)	
- Tocilizumab only	42 (6.1%)	27 (4.5%)	15 (17.2%)	

PCPF, post-COVID pulmonary fibrosis. Bold and * indicate *P* < 0.05.

Treatment regimens were subdivided into detailed categories, including steroid combinations and switching patterns, as well as antiviral and immunomodulator use. Among steroid regimens, PCPF patients more frequently underwent dexamethasone switching compared with non-PCPF patients (46.0% vs. 30.1%), whereas the use of dexamethasone only (21.8% vs. 31.3%) or no steroids (11.5% vs. 20.8%) was less common (overall *p* = 0.008). Antiviral use patterns were comparable between groups, with remdesivir being the predominant agent (64.4% vs. 62.9%, overall *p* = 0.255). By contrast, no switching or combination regimens were observed for immunomodulators (Table 3). In addition, the distribution of combined use of the three major medication classes and the severity-stratified combinations are presented in Supplementary Figures 1, 2, respectively.

### Risk factors associated with the development of post-COVID lung fibrosis

We performed both univariate and multivariate logistic regression analyses to identify independent risk factors associated with the development of PCPF (Table 4).

**TABLE 4:** Univariate and multivariate logistic regression analyses for risk factors associated with PCPF.

	Univariate analysis		Multivariate analysis		
Variable	Odd ratio	*P*-value	Odd ratio	*P*-value	GVIF
Age (years)	0.992 (0.978–1.007)	0.297	0.977 (0.954–1.001)	0.058	1.165
Sex (male)	1.267 (0.800–2.034)	0.319	0.825 (0.415–1.636)	0.581	1.145
Height (cm)	1.003 (0.985–1.026)	0.747	Correlated with BMI		
Weight (kg)	**1.018 (1.003–1.035)**	**0.020**	Correlated with BMI		
BMI (kg/m^2^)	1.003 (0.994–1.009)	0.413	1.014 (1.000–1.032)	0.055	1.101
Never smoker	1.160 (0.659–2.169)	0.622	0.705 (0.284–1.842)	0.460	1.146
Myocardial infarction	0.776 (0.182–2.276)	0.683	0.634 (0.085–2.853)	0.597	1.098
Congestive heart failure	1.011 (0.583–1.695)	0.967	0.976 (0.417–2.200)	0.954	1.233
Peripheral vascular disease	1.593 (0.779–3.038)	0.177	1.724 (0.645–4.383)	0.262	1.149
Cerebrovascular disease	1.091 (0.567–1.974)	0.783	0.971 (0.347–2.561)	0.954	1.273
Dementia	0.619 (0.182–1.585)	0.372	0.618 (0.096–2.843)	0.570	1.279
Chronic pulmonary disease	1.053 (0.623–1.735)	0.842	1.122 (0.535–2.296)	0.756	1.107
Rheumatic disease	0.644 (0.102–2.264)	0.559	0.251 (0.012–1.584)	0.223	1.052
Peptic ulcer disease	1.410 (0.807–2.382)	0.212	1.638 (0.752–3.476)	0.204	1.123
Mild liver disease	1.232 (0.663–2.177)	0.489	1.601 (0.662–3.689)	0.280	1.137
Diabetes without chronic complication	0.910 (0.546–1.481)	0.709	1.072 (0.506–2.215)	0.852	1.119
Diabetes with chronic complication	0.794 (0.357–1.580)	0.538	0.498 (0.155–1.487)	0.223	1.297
Paraplegia and hemiplegia	0.929 (0.270–2.448)	0.894	0.771 (0.082–4.783)	0.796	1.243
Renal disease	1.400 (0.810–2.349)	0.213	**3.287 (1.260–8.580)**	0.014*	**1.447**
Any malignancy†	**0.588 (0.369–0.936)**	**0.025**	0.830 (0.385–1.762)	0.629	1.285
Moderate or severe liver disease	0.812 (0.127–2.924)	0.784	0.882 (0.107–4.878)	0.894	1.159
Metastatic solid tumor	0.505 (0.254–0.926)	0.037	0.426 (0.151–1.082)	0.086	1.160
Severity (ref. mild to moderate)					1.105
Severity (severe)	0.868 (0.047–4.669)	0.784	1.007 (0.047–7.233)	0.995	
Severity (critical)	**2.745 (1.611–4.927)**	**0.037**	1.264 (0.538–3.143)	0.599	
COT	**1.991 (1.247–3.250)**	**0.005**	Correlated with severity		
HFNC	**2.419 (1.457–4.185)**	**0.001**	Correlated with severity		
IMV	**3.325 (1.933–5.610)**	**< 0.001**	Correlated with severity		
ECMO	**22.185 (5.016–153.171)**	**< 0.001**	Correlated with severity		
Hemoglobin (g/dL)	**1.189 (1.075–1.319)**	**0.001**	**1.194 (1.032–1.387)**	0.019*	**1.226**
WBC (10^3^/μL)	0.981 (0.934–1.023)	0.417	0.941 (0.866–1.010)	0.126	1.187
Platelet (10^3^/μL)	1.000 (0.998–1.002)	0.984	1.000 (0.997–1.003)	0.999	1.179
CRP (mg/dL)	**1.004 (1.001–1.007)**	**0.016**	**1.005 (1.001–1.009)**	0.022*	**1.136**
AST (IU/L)	1.001 (0.999–1.002)	0.359	Correlated with ALT		
ALT (IU/L)	1.001 (0.998–1.003)	0.450	1.000 (0.997–1.002)	0.949	1.040
Total protein (g/dL)	0.819 (0.619–1.080)	0.158	0.925 (0.622–1.371)	0.696	1.163
Albumin (g/dL)	0.920 (0.679–1.274)	0.602	1.166 (0.749–1.962)	0.527	1.195
Creatinine (mg/dL)	0.997 (0.813–1.170)	0.970	0.880 (0.673–1.121)	0.315	1.282
Concurrent bacterial pneumonia	**1.771 (1.072–2.870)**	**0.022**	0.913 (0.428–1.894)	0.810	1.161
Steroid	**2.022 (1.063–4.265)**	**0.045**	Correlated with each corticosteroid agents		
Dexamethasone	1.325 (0.828–2.165)	0.250	1.183 (0.576–2.492)	0.651	1.150
Prednisolone	**2.631 (1.656–4.259)**	**< 0.001**	1.046 (0.435–2.437)	0.919	1.388
Methylprednisolone	**3.109 (1.939–4.960)**	**< 0.001**	2.275 (0.945–5.703)	0.071	1.494
Hydrocortisone	1.128 (0.575–2.064)	0.710	0.694 (0.263–1.672)	0.435	1.126
Remdesivir	1.043 (0.657–1.683)	0.860	**0.359 (0.176–0.734)**	0.005*	**1.115**
Nirmatrelvir_ritonavir	0.859 (0.201–2.526)	0.807	1.016 (0.139–4.740)	0.985	1.096
Molnupiravir	0.000 (0.000–999.999)	0.983	0.000 (0.000–999.999)	0.988	1.000
Baricitinib	**4.970 (1.896–12.394)**	**0.001**	**5.633 (1.624–19.548)**	0.006*	**1.153**
Tocilizumab	**4.429 (2.205–8.621)**	**< 0.001**	2.036 (0.721–5.615)	0.172	1.259

PCPF, post-COVID pulmonary fibrosis; GVIF, generalized variance inflation factor; BMI, body mass index; COT, conventional oxygen therapy; HFNC, high-flow nasal cannula; IMV, invasive mechanical ventilation; ECMO, extracorporeal membrane oxygenation; WBC, white blood cells; CRP, C-reactive protein, AST, aspartate aminotransferase; ALT, alanine aminotransferase. Bold and * indicate *P* < 0.05.

† Including lymphoma and leukemia, except malignant neoplasm of the skin.

In the univariate logistic regression analysis, several variables were significantly associated with the development of PCPF. Patients with higher body weight were more likely to develop PCPF (odds ratio [OR]: 1.018, 95% confidence interval [CI]: 1.003–1.035, *p* = 0.020). The presence of solid and metastatic tumors was inversely associated with fibrosis (solid tumors: OR: 0.588, 95% CI: 0.369–0.936, *p* = 0.025; metastatic tumors: OR: 0.505, 95% CI: 0.254–0.926, *p* = 0.037). Clinical severity played a significant role: critically ill patients (OR: 2.745, 95% CI: 1.611–4.927, *p* = 0.037) and those requiring oxygen support, including COT (OR: 1.991, 95% CI: 1.247–3.250, *p* = 0.005), HFNC (OR: 2.419, 95% CI: 1.457–4.185, *p* = 0.001), mechanical ventilation (OR: 3.325, 95% CI: 1.933–5.610, *p* < 0.001), and ECMO (OR: 22.185, 95% CI: 5.016–153.171, *p* < 0.001), were more likely to develop fibrosis. Among laboratory parameters, elevated hemoglobin (OR: 1.189, 95% CI: 1.075–1.319, *p* = 0.001) and CRP (OR: 1.004, 95% CI: 1.001–1.007, *p* = 0.016) were associated with PCPF development. In addition, concurrent bacterial pneumonia was significantly associated with the presence of PCPF (OR 1.771, 95% CI 1.072–2.870, *p* = 0.022). The use of systemic steroids (OR: 2.022, 95% CI: 1.063–4.265, *p* = 0.045), particularly prednisolone (OR: 2.631, 95% CI: 1.656–4.259, *p* < 0.001) and methylprednisolone (OR: 3.109, 95% CI: 1.939–4.960, *p* < 0.001), showed significant associations with PCPF. Additionally, the use of baricitinib (OR: 4.970, 95% CI: 1.896–12.394, *p* = 0.001) and tocilizumab (OR: 4.429, 95% CI: 2.205–8.621, *p* < 0.001) was significantly higher among patients who developed fibrosis than in patients who did not.

In the multivariate model, pre-existing renal disease was independently associated with increased risk of PCPF (adjusted OR [aOR] 3.287; 95% CI: 1.260–8.580; *p* = 0.014). Laboratory data in the acute phase revealed that higher hemoglobin levels (aOR: 1.194, 95% CI: 1.032–1.387, *p* = 0.019) and elevated CRP (aOR: 1.005, 95% CI: 1.001–1.009, *p* = 0.022) were independently associated with an increased risk of PCPF. Among therapeutic agents, the use of baricitinib was associated with the development of PCPF (aOR: 5.633, 95% CI: 1.624–19.548, *p* = 0.006), while the use of remdesivir was associated with a reduced risk (aOR: 0.359, 95% CI: 0.176–0.734, *p* = 0.005).

Interaction analyses were performed to evaluate potential drug–drug interactions among remdesivir, dexamethasone, and baricitinib. However, no statistically significant associations were identified in the two pairwise comparisons (Δ Deviance = 7.135, Δdf = 3, *p* = 0.068; Supplementary Table), and analysis of the three-drug combinations was not feasible due to the limited sample size (Supplementary Figures 1, 2).

## Discussion

In this multicenter retrospective cohort study, 12.6% of the hospitalized adult patients developed PCPF, based on follow-up chest CT imaging. This observation is consistent with prior studies, which reported that fibrotic pulmonary sequelae occur in approximately 10–40% of patients with COVID-19, particularly those who experienced severe or critical illness necessitating advanced respiratory support (Myall et al., 2021; Yoon and Uh, 2022). Our analysis identified several factors associated with PCPF, including pre-existing renal disease, elevated inflammatory markers, intensive respiratory support, and specific pharmacologic exposures.

Chronic kidney disease (CKD) was identified as a strong independent risk factor for PCPF. This may be attributable to systemic inflammation, endothelial dysfunction, and lung–kidney organ crosstalk, mechanisms by which CKD has been linked to increased susceptibility to restrictive and fibrotic lung conditions (Bollenbecker et al., 2022). Moreover, CKD has been linked with impaired pulmonary physiology via fluid overload, acid–base disturbances, and altered vascular tone, which can predispose the lung to fibrotic remodeling (Gembillo et al., 2023). From this perspective, renal disease has also been associated with increased mortality among patients with COVID-19 (Choi et al., 2021). Despite limited studies in COVID-19 contexts, this association suggests that COVID-19 patients with CKD require vigilant respiratory monitoring.

We observed that patients with PCPF had significantly higher hemoglobin levels and CRP during the acute phase of COVID-19, both of which were independently associated with fibrosis in the multivariate analysis. The association between hemoglobin levels and PCPF is not well established. In a previous study, lower hemoglobin concentrations were associated with an increased risk of PCPF (Li et al., 2022). In contrast, our findings suggest that higher hemoglobin levels may be linked to fibrosis development. Elevated hemoglobin is generally regarded as a compensatory response to chronic hypoxemia. Therefore, our findings suggest that a prolonged or more severe hypoxic burden during the acute phase of COVID-19 promotes epithelial–mesenchymal transition and extracellular matrix remodeling, key processes implicated in the pathogenesis of hypoxia-induced pulmonary fibrosis (Zhou et al., 2009). However, this hypothesis warrants further investigation in prospective studies. Elevated CRP is a well-established marker of systemic inflammation and is involved in the pathogenesis of pulmonary fibrosis by promoting alveolar epithelial injury and fibroblast activation through interleukin-6 (IL-6) and transforming growth factor-beta (TGF-β) signaling pathways (George et al., 2020; Mylvaganam et al., 2021). These results support existing evidence linking inflammation and hypoxia to post-viral fibrotic lung disease.

Disease severity was another strong predictor of PCPF, with patients requiring mechanical ventilation or ECMO exhibiting a markedly higher incidence of fibrosis. These findings reinforce the hypothesis that ventilator-induced lung injury, oxygen toxicity, and secondary infections contribute significantly to fibroproliferative lung damage in COVID-19 patients (Michalski et al., 2022). This is further supported by reports indicating that post-ARDS fibrotic changes observed in COVID-19 closely resemble those seen in non-COVID ARDS patients (Barbeta et al., 2022). Recent studies have also suggested that SARS-CoV-2 infection may induce hypoxia-related pathways through increased expression of HIF-1α, further promoting pulmonary fibrogenesis (Tian et al., 2021; Devaux and Lagier, 2023).

In our analysis, concurrent bacterial pneumonia was significantly associated with PCPF in univariate analysis, but this relationship did not persist in the multivariate model after adjustment for other covariates. This suggests that the observed association may be confounded by underlying disease severity or immunosuppressive treatment exposure rather than a direct causal effect. Previous studies have reported that bacterial superinfection is common in severe COVID-19 and may contribute to prolonged inflammation and adverse pulmonary outcomes (Langford et al., 2020; Garcia-Vidal et al., 2021). However, the direct role of bacterial pneumonia in the development of chronic fibrotic sequelae remains uncertain, with some cohorts failing to demonstrate an independent effect once adjusted for baseline severity and treatment factors (Myall et al., 2021; Wu et al., 2021). Taken together, these findings highlight the need for prospective studies to clarify whether concurrent bacterial pneumonia truly contributes to post-COVID fibrosis, or whether it simply reflects patients with more severe acute disease trajectories.

The current study also revealed that remdesivir use was associated with a significantly lower risk of fibrosis. Recent animal studies have shown that remdesivir attenuates lung fibrosis in bleomycin-induced mouse models by inhibiting TGF-β1-mediated pathways (Li et al., 2021). To date, no clinical studies have specifically evaluated the effect of remdesivir on the development of lung fibrosis in patients with COVID-19. Remdesivir is recommended for patients with severe COVID-19, but not for those with critical illness requiring mechanical ventilation or ECMO, due to its limited efficacy in critical disease (Beigel et al., 2020). In addition, the increased risk of fibrosis among patients requiring mechanical ventilation or ECMO may, at least in part, reflect the intense inflammatory response and immune dysregulation characteristic of severe COVID-19 (Zhou et al., 2025). Therefore, the observed association between remdesivir use and reduced risk of fibrosis may be subject to confounding by indication. To minimize this potential bias, our analysis adjusted for key clinical variables, including disease severity, age, and underlying comorbidities. Our findings suggest a possible long-term benefit in preventing fibrotic lung changes. Nevertheless, further investigation is needed to confirm these findings.

Conversely, the use of baricitinib was strongly associated with the development of fibrosis. While baricitinib is known to improve survival and reduce progression to mechanical ventilation in severe COVID-19 (Ely et al., 2022), its association with fibrosis also reflects confounding by indication, as it is often reserved for patients with hyperinflammatory states who are already at high risk of fibrotic complications. The association between baricitinib and PCPF remains unclear. Although clinical data are limited, preclinical studies have demonstrated that baricitinib attenuates lung fibrosis in mouse models by inhibiting the JAK/STAT and TGF-β signaling pathways (Gu et al., 2023). Therefore, further research is needed to elucidate the potential impact of baricitinib on fibrotic outcomes in humans.

Although, our study did not examine potential drug–drug interactions, and therefore the impact of clinically used COVID-19 treatment combinations on the development of PCPF remains uncertain. Previous reports have suggested possible synergistic effects of certain antiviral and immunomodulatory agents, such as remdesivir combined with baricitinib (Kalil et al., 2021), dexamethasone–baricitinib–remdesivir triple therapy (Yasuda et al., 2022; Mozaffari et al., 2025), or repurposed agents including fluoxetine and amiodarone (Schloer et al., 2021; Schreiber et al., 2022). Given our retrospective design and limited sample size, further stratification by multiple drug regimens was not feasible and would have resulted in sparse cells and unstable estimates. Future prospective studies with larger populations are needed to clarify whether specific drug combinations modify the risk of post-COVID pulmonary fibrosis.

Our study has several strengths. We analyzed a large, multicenter cohort using consistent inclusion criteria and expert-confirmed CT diagnoses. A comprehensive set of covariates, including inflammatory markers, medications, and respiratory interventions, was adjusted for in the multivariable analysis. Notably, this is the first human study to suggest a potential protective role for remdesivir against post-COVID fibrosis, bridging preclinical evidence with real-world outcomes.

Nevertheless, this study has some limitations. First, its retrospective design carries an inherent risk of residual confounding, particularly with respect to treatment selection. The associations observed with antiviral or immunomodulatory agents should be interpreted cautiously due to indication bias. Second, imaging interpretation was not centrally standardized, and quantitative fibrosis scoring was not applied. Third, because patients with pre-existing lung fibrosis on pre-COVID chest CT scans were excluded, those receiving antifibrotic agents such as pirfenidone or nintedanib were not included, leaving the potential effect of antifibrotic therapy on PCPF unclear. Lastly, this study did not include data on SARS-CoV-2 variants (Sohn et al., 2022; Seo et al., 2023). Since different SARS-CoV-2 variants predominated during various phases of the pandemic, and each variant has been associated with distinct clinical outcomes, the inability to identify the dominant strain at the time of infection—due to the limitations of the standard RT-PCR used in this study—may have introduced potential bias in interpreting disease progression and fibrotic outcomes. Furthermore, as repeated PCR testing was not performed, we could not directly assess changes in viral load during the recovery period, which may have influenced the interpretation of post-COVID radiologic findings.

In conclusion, our study demonstrates that PCPF remains a relevant long-term complication in a subset of COVID-19 survivors. Elevated CRP and hemoglobin during the acute phase are potential early indicators. Remdesivir use was associated with a lower risk of fibrosis, while baricitinib was associated with a higher risk, reflecting a possible confounding by disease severity. Prospective studies are warranted to validate these findings and inform post-COVID management strategies.

## Data Availability

The raw data supporting the conclusions of this article will be made available by the authors, without undue reservation.
