# Supplementary material for: Impact of COVID-19 therapeutics on the development of post-infectious lung fibrosis
Source: Front Microbiol. 2025 Nov 6;16:1677734. doi: 10.3389/fmicb.2025.1677734 (PMC12631368; doi:10.3389/fmicb.2025.1677734)
Supplement: Supplementary file 1 [file Table_1.docx]

**Supplemenatary materials**

Supplementary Figure 1. Distribution of steroid, antiviral, and immunomodulator combinations by PCPF status


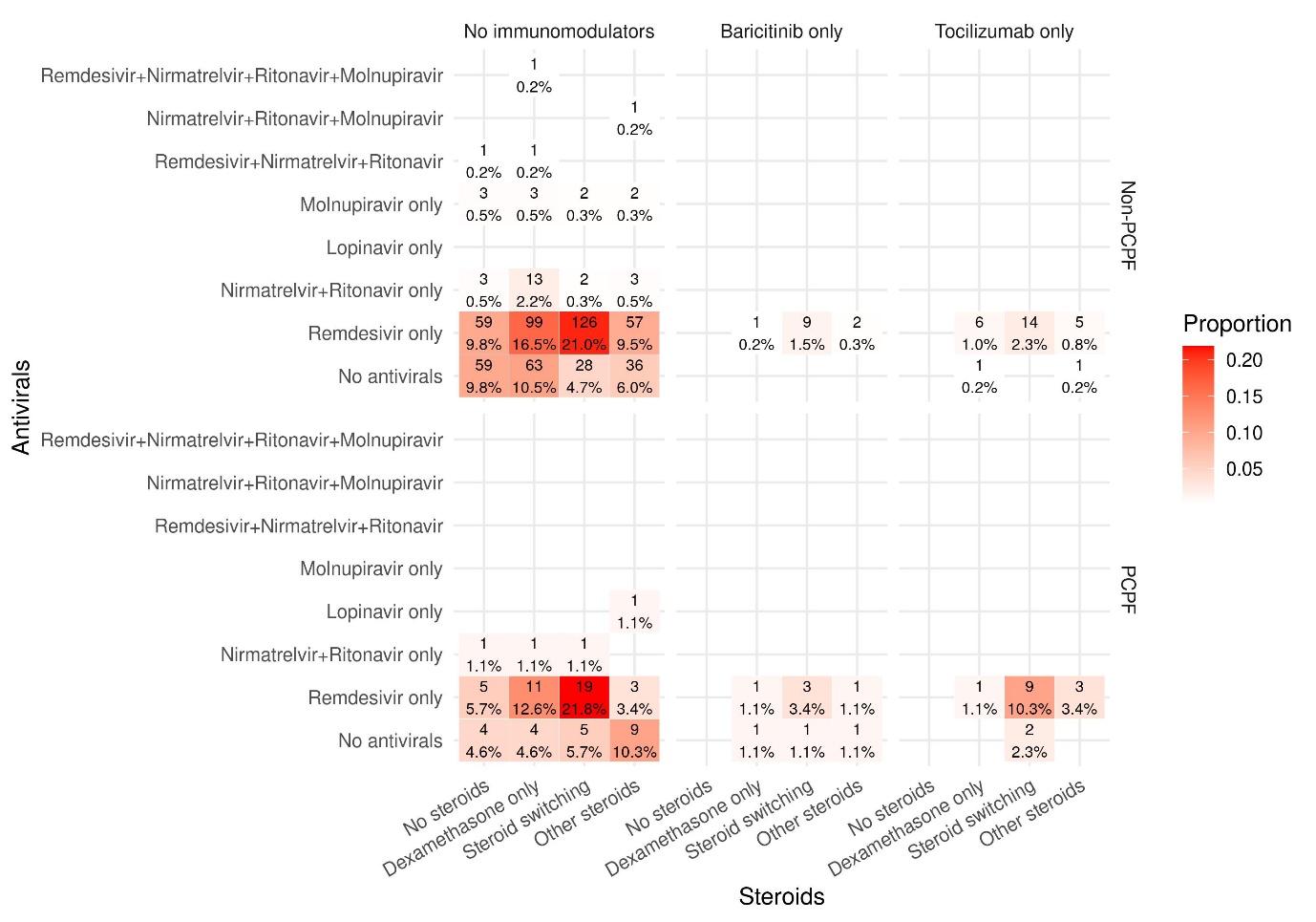


“Steroids switching” was defined as regimen changes that included dexamethasone.

Supplementary Figure 2. Distribution of steroid, antiviral, and immunomodulator combinations by PCPF status stratified by COVID-19 severity


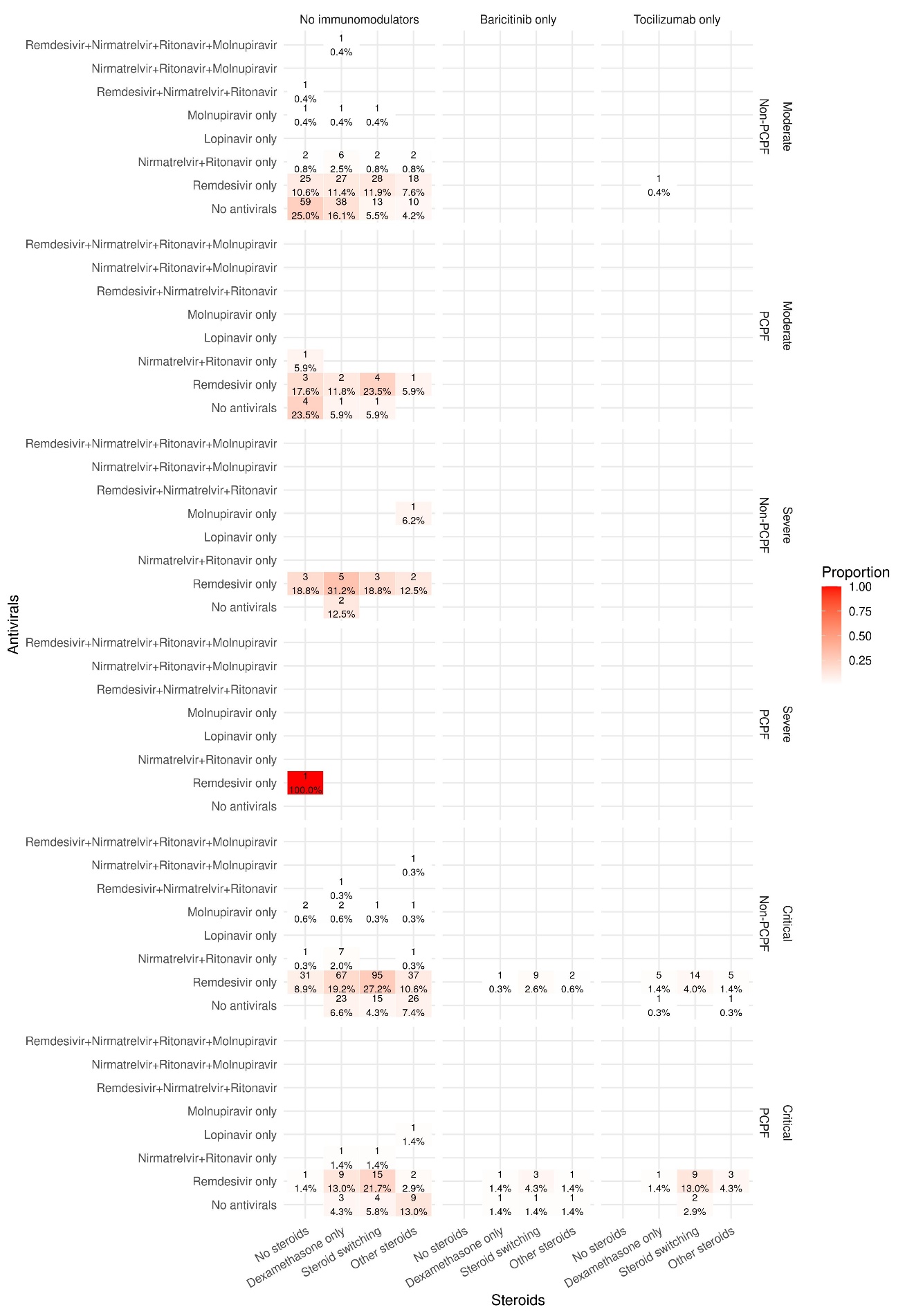


"Steroids switching” was defined as regimen changes that included dexamethasone.

Supplementary Table. Interaction analysis for drug–drug interactions in in PCPF

|  | **Multivariate model 1** | | **Multivariate model 2** | |
| --- | --- | --- | --- | --- |
|  | Baseline effect  (without interaction) | | Synergic effect  (with interactions-2 pairwise) | |
|  | OR | P-value | OR | P-value |
| Weight | 1.023 (1.002–1.044) | 0.023 | 1.024 (1.003–1.045) | 0.020 |
| Any malignancy | 0.879 (0.454–1.684) | 0.698 | 0.936 (0.475–1.829) | 0.846 |
| Metastatic solid tumor | 0.409 (0.161–0.944) | 0.045 | 0.397 (0.155–0.926) | 0.041 |
| Severity-severe) | 0.816 (0.042–4.985) | 0.853 | 0.813 (0.042–4.992) | 0.851 |
| Severity-critical) | 1.510 (0.709–3.454) | 0.304 | 1.359 (0.629–3.139) | 0.450 |
| Hemoglobin (g/dL) | 1.171 (1.040–1.322) | 0.010 | 1.188 (1.053–1.346) | 0.006 |
| CRP (mg/dL) | 1.004 (1.000–1.007) | 0.048 | 1.004 (1.000–1.007) | 0.030 |
| Concurrent bacterial pneumonia | 1.122 (0.594–2.075) | 0.718 | 1.094 (0.570–2.055) | 0.782 |
| Remdesivir | 0.412 (0.223–0.768) | 0.005 | 0.272 (0.091–0.796) | 0.017 |
| Dexamethasone | 1.341 (0.721–2.569) | 0.363 | 0.808 (0.269–2.450) | 0.703 |
| Baricitinib | 3.265 (1.124–9.121) | 0.025 | 999.999 (0.000–999.999) | 0.981 |
| Remdesivir:Dexamethasone |  |  | 2.397 (0.611–9.707) | 0.212 |
| Remdesivir:Baricitinib |  |  | 0.000 (0.000–999.999) | 0.983 |
| Dexamethasone:Baricitinib |  |  | 0.201 (0.012–5.714) | 0.271 |

PCPF, post-COVID pulmonary fibrosis; CRP, C-reactive protein.

Comparison between Model 1 (without interactions; residual deviance = 337.91, df = 467) and Model 2 (with interactions; residual deviance = 330.77, df = 464) indicated no statistically significant improvement in model fit (ΔDeviance = 7.135, Δdf = 3, p = 0.068). Analysis of the three-drug (remdesivir dexamethasone, and baricitinib) combinations was not feasible because of the limited sample size.
